# Mediating Effect of Sports Participation on the Relationship between Health Perceptions and Health Promoting Behavior in Adolescents

**DOI:** 10.3390/ijerph17186744

**Published:** 2020-09-16

**Authors:** Seung-Man Lee, Hyun-Chul Jeong, Wi-Young So, Hyun-Su Youn

**Affiliations:** 1 Department of Physical Education, Jingwan High School, Seoul-si 03302, Korea; lsm14pe@gmail.com; 2 Department of Physical Education, Jeonbuk National University High School, Jeollabuk-do 54896, Korea; 01086445918@hanmail.net; 3 Sports and Health Care Major, College of Humanities and Arts, Korea National University of Transportation, Chungju-si 27469, Korea; 4 Department of Physical Education, College of Education, WonKwang University, Iksan-si 54538, Korea

**Keywords:** adolescent, health perceptions, health promoting behavior, sports participation, mediating effect

## Abstract

The aim of this study was to verify the structural relationship between health perceptions, sports participation, and health promoting behavior in adolescents. A total of 507 adolescents living in Seoul, Republic of Korea, in 2020, participated in this study. This study was conducted using a preliminary survey and a main survey. In the preliminary survey, the reliability and validity of the scales used in this study were analyzed, and in the main survey, the relationships between individual variables were verified. Specifically, descriptive statistical analysis, path analysis, and mediating effect analysis were conducted in the main survey. The results of the study are as follows: first, health perceptions were found to have a positive effect on sports participation (*p* < 0.001). Furthermore, health perceptions were found to have no direct effect on health promoting behavior (*p* = 0.554), while sports participation was found to have a positive effect on health promoting behavior (*p* < 0.001). Additionally, sports participation completely mediated the relationship between health perceptions and health promoting behavior. Based on the results of this study, suggestions are presented on how to enhance health perceptions in adolescents who are in a critical period for forming healthy life habits, and to prepare measures to encourage sports participation.

## 1. Introduction

As of 2020, the world is facing the coronavirus disease (COVID-19) pandemic. Scientists predict that the COVID-19 transmission may be at controllable levels over time, but that increasingly more novel viruses will be encountered. The outbreak of new viral infections is accelerating the development of treatment and vaccines at the national global level. People are responding to the new viral infections by strengthening environmental and personal hygiene and enhancing their individual immunity. In addition, people’s interest in immunity-related health and related desires is increasing. However, as acquiring healthy life habits takes time, it is necessary to encourage adolescents to develop conscious health perceptions and healthy life habits.

While emphasizing that the formation of healthy habits in adolescence is the foundation of lifelong health, it is also necessary to improve adolescents’ health interests in various ways. This approach addresses the concept of health perceptions that are likely to be an important factor for determining future health behavior in adolescents. Health perceptions refer to a subjective process in which individuals are conscious of external stimuli related to health through sensory organs [1]. However, individuals do not perceive health in the same way, perceptions of health may differ based on how the person views and interprets facts [2]. Therefore, previous studies have claimed that the world perceived by each individual is different in similar situations, and the resulting behavior initiated by each individual is different [3,4].

Such health perceptions in adolescents can be naturally linked to health promoting behaviors and habits. Health promoting behavior involves an individual’s perception of the importance of health for himself/herself and of engaging in preventive behaviors for promoting health. Furthermore, it refers to improving health care skills by changing life habits through knowledge, attitudes, and behaviors about health [4]. In addition, because health promoting behavior can reduce the cost of personal health care and is associated with an extended lifespan and improving quality of life, it can bring benefits not only at an individual but also a national level.

The relationship between health perceptions and health promoting behavior has been reported in previous studies. The findings suggest that higher health perceptions in middle and high school students were associated with better health behavior [5]. Furthermore, an investigation into the relationship between perceived health status and other variables found that people with higher social support evaluated their health status more positively [6]. In addition, the importance of health perceptions have been emphasized because they determine the attitudes and behavioral characteristics of an individual or a group, and have a direct or indirect effect on health [7]. Moreover, a study described that health promoting behavior and health perceptions could be enhanced through personalized health care customized to individual characteristics, and adapted to school life, thereby forming healthy life habits and enabling access to health care [7].

Furthermore, it focused on the possibility and role of sports participation in the “process” where health perceptions in adolescents affect health promoting behavior. Sports participation is a social behavior initiated in connection with various sports phenomena and can be divided into behavioral, cognitive, affective, and social participation [8]. Physical activity habits formed through regular sports participation can be regarded as important variables that are highly likely to maintain and promote health, along with eating habits and life habits. Previous research [9,10,11,12] has reported a positive causal link between health perceptions and sports participation, this suggests that it can be predicted that adolescents’ health perceptions could serve as an antecedent variable affecting sports participation. In addition, considering the results of previous studies [13,14,15] regarding the relationship between sports participation and health promoting behavior, it can be assumed that sports participation may be related to health promoting behavior. The results of the aforementioned studies suggest that adolescents’ health perceptions may affect health promoting behavior, and that sports participation may act as an explanatory variable in the relationship between health perceptions and health promoting behavior. However, since previous studies mainly involved adults, including older adults, it is difficult to generalize these results to adolescents. In addition, previous studies are limited in that they sporadically report only some of the variables in the relationships among health perceptions, sports participation, and health promoting behavior. In order to overcome these limitations, it is necessary to verify the effects of health perceptions and sports participation on improving health promoting behavior in adolescents, using an appropriate study design.

**Figure 1:**
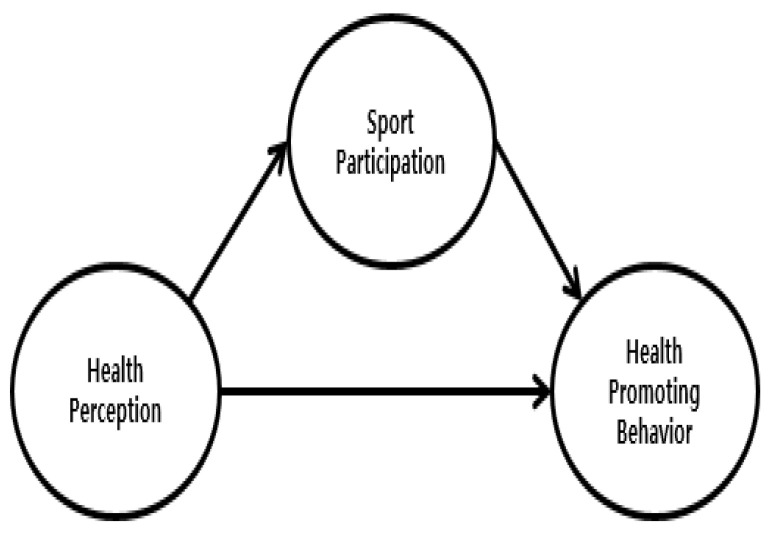
Hypothesis model.

**Figure 2:**
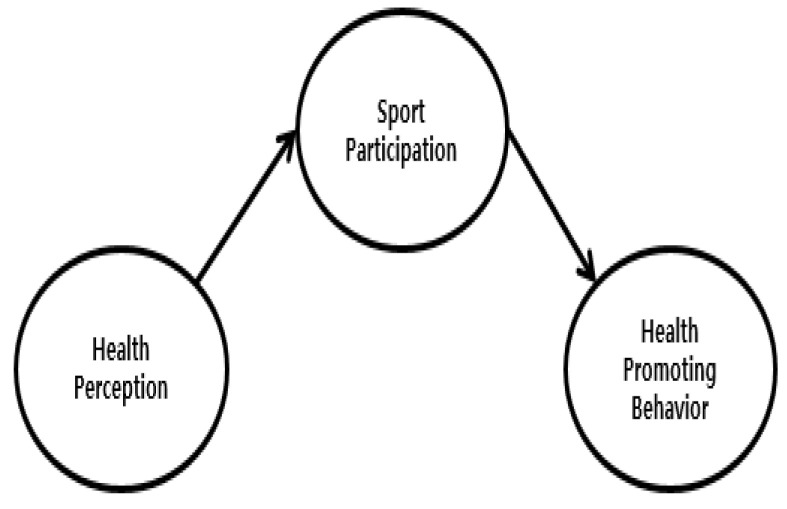
Competition model.

Reflecting on these points, this study aims to clarify the structural relationship between health perception, health promoting behavior, and sports participation, and to investigate the mediating effect of sports participation in the relationship between health perceptions and health promoting behavior. In order to clarify the aims of this study, the research hypotheses were established as follows: first, health perceptions will have a positive effect on sports participation (H1). Second, health perceptions will have a positive effect on health promoting behavior (H2). Third, sports participation will have a positive effect on health promoting behavior (H3). Fourth, sport participation will have a mediating effect on the relationship between health perceptions and health promoting behavior (H4). Specifically, sports participation will partly mediate the relationship between health perceptions and health promoting behavior (hypothesis model illustrated in Figure 1) or will fully mediate the relationship between health perception and health promoting behavior (competition model illustrated in Figure 2).

## 2. Methods

### 2.1. Participants

The population of this study was adolescents living in the Republic of Korea in July 2020. A total of 550 Korean adolescents were recruited to participate in this study using convenience sampling, a nonprobability sampling method, and were surveyed using Google Forms to collect the survey. The use of Google Forms could raise concerns about the accuracy of the answers. However, due to the practice of “social distancing” caused by COVID-19, online questionnaires were necessary. A total of 507 survey responses were used in the study, 43 surveys with incomplete information, which were judged to be inadequate for the purpose of data analysis, were excluded. This study was conducted after obtaining ethical approval from the Institutional Review Board of WonKwang University (WKIRB-202007-SB-034). The general characteristics of the participants are shown in Table 1.

### 2.2. Instruments

This study used scales that were deemed suitable for the purpose of this study selected from scales used in previous studies. The general characteristics of the participants were measured using two items regarding school level and gender on a nominal scale. Health perceptions were measured using a scale that was based on the health perception scale developed by Ware [2] and was verified for its reliability and validity by Kim and Choi [1], Kim, Kim, and Sok [7], and Kwon [16]. Specifically, this scale consists of 4 subscales, with a total of 20 items, measuring the importance of health, health interest, confidence in health recovery, and health concern. Health promoting behavior was measured using a Korean version of the Health-Promoting Lifestyle Profile-II (HPLP-II) originally developed by Walker, Sechrist, and Pender [4], which was verified for its reliability and validity by Kim [13], Kim [17], and Kang [18]. Specifically, this scale consists of 5 subscales, with a total of 20 items, measuring personal hygiene and life habits, nutrition and exercise management, eating habit management, stress management, and health responsibility. Sports participation was measured using a tool based on a classification model for sports participation developed by Snyder and Spreitzer [19], which was verified for its reliability and validity by Lee [20], Lee and Lee [21,22]. The scale consists of 3 subscales with a total of 20 items, measuring cognitive participation, behavioral participation, and affective participation. Health perceptions, health promoting behavior, and sports participation were independently scored on a 5-point Likert scale, ranging from “strongly agree” (5 points) to “not at all” (1 point).

### 2.3. Reliability and Validity of Instruments

A preliminary survey was conducted to verify the reliability and validity of the instruments. The reliability was verified using Cronbach’s α, which tests the internal consistency of the items, and confirmatory factor analysis was performed to test validity. Reliability is related to how consistently and accurately a method measures something and indicates the accuracy of the measurement [23]. The reliability for each variable used in this study is shown in Table 2.

Generally, Cronbach’s α cannot be judged to be unreliable until it reaches 0.7 or higher, but some scholars have argued that this scale’s reliability can be ensured even if it reaches 0.6 or 0.5 or higher [23]. The Cronbach’s α values of the observed variables used to measure health perceptions were between 0.526 and 0.787, all of which were more than the reference value of 0.500, indicating that the internal consistency reliability was appropriate [23]. In addition, “Alpha If Item Deleted” eliminated items (health interest #5, confidence in health recovery #1, health concern #4) higher than the total Cronbach’s α after deleting respective items were deleted.

The Cronbach’s α values of the overserved variables used to measure health promoting behavior ranged from 0.359 to 0.699, and the variable (personal hygiene and life habits) that did not show the desired internal consistency reliability was deleted. All other variables were evaluated as having an appropriate internal consistency reliability with a Cronbach’s α reference value of 0.500 or above. In addition, items with a Cronbach’s α value higher than the total Cronbach’s α (eating habit management #1) after deleting respective items were deleted.

In addition, the Cronbach’s α values of the variables used to measure sports participation ranged from 0.811 to 0.917, and the values for all variables were above the reference value of 0.5, indicating that the internal consistency reliability was at an appropriate level. Items with a Cronbach’s α higher than the total Cronbach’s α (cognitive participation #4, behavioral participation #3, and affective participation #1) after deleting respective items, were deleted.

Meanwhile, confirmatory factor analysis was used to test convergent validity, nomological validity, and discriminant validity of the scales. The goodness-of-fit indexes in confirmatory factor analysis were tested for incremental fit index through Incremental Fit Index (IFI) and Comparative Fit Index (CFI), and for absolute fit index through Chi-Square/Degrees of Freedom (x^2^/DF), Root Mean Square Error of Approximation (RMSEA), Goodness of Fit Index (GFI), and Root Mean square Residual (RMR). The results are shown in Table 3.

However, some indexes were found to be below the reference value, and some items (health concern) were removed based on the squared multiple correlation (SMC) value. Consequently, the goodness-of-fit of the revised model was found to be good overall. The detailed goodness-of-fit indexes in the confirmatory factor analysis of the proposed and revised models are shown in Table 3.

In addition, the validity of the model was tested through confirmatory factor analysis and results are presented in Table 4. The convergent validity was verified using three methods: standardized regression coefficient, average variance extracted, and construct reliability. The standardized regression coefficients for all variables ranged from 0.555 to 0.946, and the significance (critical ratio) was 1.965 or higher. In addition, the construct reliability was found to be between 0.547 and 0.985, and the average variance extracted was between 0.917 and 0.955, indicating that the convergent validity was appropriate.

The nomological validity was tested. This study predicted the relationship between constructs in a positive (+) direction, and the main relationship between latent variables showed a significant positive (+) relationship (Table 4), indicating that the nomological validity was secured.

The discriminant validity was verified by comparing the correlations between the constructs, and the average variance extracted (Table 5). The squared value of the correlation coefficient for “health perception ↔ health promoting behavior” was obtained, and the highest correlation was 0.397, which was lower than the average variance extracted of health perception (0.951) and health promoting behavior (0.955), indicating that the discriminant validity between the variables was secured.

### 2.4. Procedure and Data Analysis

The data were collected through two online surveys (a preliminary survey and a main survey) using Google Forms. The preliminary survey was conducted on 200 Korean adolescents in 2020, and a total of 180 survey responses, after excluding 20 survey responses with incomplete information, were finally used for analysis. The main survey was conducted on 350 Korean adolescents in 2020, and 327 survey responses, excluding 23 survey responses with incomplete information, were finally used for analysis

The data collected were analyzed using SPSS and AMOS 18.0 program (IBM Corp., Armonk, NY, USA). The detailed analyses were as follows. First, frequency analysis was performed to examine the general characteristics of the participants (see Section 2.1). Second, the reliability of the tools used in this study was tested using Cronbach’s α (see Section 2.3). Third, confirmatory factor analysis was performed to test the validity of the tools, and then convergent, nomological, and discriminant validities of the tools were tested (see Section 2.3). Fourth, a descriptive statistical analysis was performed to examine the perception of each variable by the participants (see Section 3.1). Fifth, the goodness-of-fit of a hypothesis model was tested to verify the structural relationship between individual variables, and then a path analysis was performed (see Section 3.2). Sixth, bootstrapping was used to verify the mediating effect of sports participation on the relationship between health perceptions and health promoting behavior in adolescents (see Section 3.3). It suggested that because it is difficult to ensure that the distribution of mediating effects is normal, 10,000 bootstrap samples generated by random sampling from raw data are to be used for parameter estimation, and a confidence interval is to be set at 95% [24]. The bootstrap method was used in accordance with the suggestions. In addition, the indirect effects of health perceptions on health promoting behavior through sports participation were examined.

## 3. Results

### 3.1. Descriptive Statistical Analysis

To investigate the descriptive statistics of the variables (health perceptions, health promoting behavior, and sports participation) used in this study, all variables and sub-variables were analyzed, and the results are shown in Table 6. The mean values were distributed between 2.58 and 3.83, and the standard deviations were distributed between 0.53 and 1.11. Then, the skewness and kurtosis were examined.

In general, it was assumed that a skewness value of <3.00 and a kurtosis value of <±10.00 are the bases of the violations of univariate normality assumptions [25,26]. The analysis results reveal that the absolute value of the skewness was distributed between 0.004 and 0.404, and the absolute value of the kurtosis was distributed between 0.047 and 1.557. These results could be evaluated as satisfying the conditions required for the normality for the structural equation model.

### 3.2. Analysis of the Relationship between the Individual Variables

The structural equation model developed in this study consisted of three latent variables: health perceptions, sports participation, and health promoting behavior, and 10 observed variables: importance of health, health interest, confidence in health recovery, nutrition and exercise management, eating habit management, stress management, health responsibility, cognitive participation, behavioral participation, and affective participation. A path analysis of the study model was performed, and the goodness-of-fit of the entire study model was determined to analyze direct and indirect effects. The results show that the goodness-of-fit of the proposed model was overall acceptable, as shown in Table 7.

The results of verifying the hypotheses that analyzed the causal relationships between the individual variables using the study model showed that hypotheses one and three were supported, but hypothesis two was rejected. The results of testing the hypotheses are shown in Table 8.

First, the results of analyzing hypothesis one (health perceptions will have a positive effect on sports participation) show that the path coefficient was 0.570 (t = 7.885), supporting the hypothesis. Second, the results of analyzing hypothesis two (health perception will have a positive effect on health promoting behavior) show that the path coefficient was 0.045 (t = 0.592), rejecting the hypothesis. Third, the results of analyzing hypothesis three (health perception will have a positive effect on health promoting behavior) show that the path coefficient was 0.749 (t = 7.159), supporting the hypothesis.

### 3.3. Mediating Effects of Sports Participation on the Relationship between Health Perceptions and Health Promoting Behavior

Analyses were performed to verify the model that explains the structural relationship between the individual variables by verifying the mediating effect of sports participation on the relationship between health perceptions and health promoting behavior in adolescents. First, we comparatively analyzed the goodness-of-fit of a partial mediation model (the hypothesis model) in which health perceptions might directly affect health promoting behavior, while also affecting health promoting behavior through sports participation. The goodness-of-fit of a complete mediation model (the competition model), in which there might be no direct path between health perceptions and health promoting behavior, and health perceptions might affect health promoting behavior through sports participation, was also analyzed. We explored the model that explained the experience data best and was the simplest. The goodness-of-fit indexes of the hypothesis model and the competition model were calculated for comparison, as shown in Table 9.

Since the complete mediation model is an embedded model in the partial mediating model, a x^2^ difference test was performed. In the x^2^ difference test, which tests the difference in the degrees of freedom between the two models, a goodness-of-fit of 5.99, which is statistically significant with α = 0.05, was revealed. However, the results of the x^2^ difference test show that the x^2^ difference between the two models was 0.286, and the difference in degrees of freedom between the two models was one, indicating that there was no statistically significant difference. A partial mediation model is selected if the results of the x^2^ difference test are statistically significant, and a complete mediation model is selected if they are not [27]. Therefore, both the complete mediation model and the competition model can be selected as the final model in this study. In other words, the direct effect of health perceptions on health promoting behavior were found to be not significant, whereas the indirect effect of health perceptions on health promoting behavior through sports participation was found to be significant.

In addition, the bootstrap method was used to test the indirect effect of sports participation in the relationship between health perceptions and health promoting behavior [24]. The bootstrap method was used to estimate the standard error of the indirect effect, which may be involved in the existing testing for mediating effects. With the bootstrap method, a confidence interval is provided, and if the confidence interval does not include 0, indirect effects are considered to be statistically significant.

As shown in Table 10, the indirect effect (*β* = 0.454, 95% bias-corrected confidence interval = 0.355–0.551) of health perceptions on health promoting behavior through sports participation was statistically significant. In other words, it was found that higher health perceptions were associated with higher sports participation, thereby leading to higher health promoting behavior.

## 4. Discussion

This study aimed to clarify the mediating effect of sports participation on the relationship between health perceptions and health promoting behavior in adolescents, and to determine the importance of sports participation in forming healthy life habits during adolescence. The principal results of this study are as follows: (1) health perceptions had a positive effect on sports participation. (2) Health perceptions had no direct effect on health promoting behavior. (3) Sports participation had a positive effect on health promoting behavior. (4) Sports participation had a complete mediating effect on the relationship between health perceptions and health promoting behavior. These findings, in comparison with the results of the previous studies, are discussed below.

First, health perceptions had a positive effect on sports participation. Previous studies regarding the relationship between sports participation and health perceptions [9,10,11,12,28] have reported that there was a positive causal relationship between the two variables, supporting the results of this study. In particular, a previous study reported that health perceptions improved with participation in exercise [11], and another study reported that sports activities had a positive effect on subjective health perceptions [1]. As shown in the results of this study and previous studies, adolescents with high health perceptions can be interpreted as actively participating in physical activities, such as sports, in order to maintain and improve their health. Accordingly, various measures should be provided to improve health perceptions in adolescence, which is a period that can lay the foundations for lifelong health education. In addition, efforts are needed to improve health perceptions, and to find and facilitate ways for adolescents to voluntarily participate in sports.

Second, health perceptions had no direct effect on health promoting behavior, but to have an indirect effect on health promoting behavior through sports participation. Previous studies have reported a positive correlation between health perceptions and health-promoting behavior, which is supported by the results of this study [3,29,30,31,32,33]. In particular, previous studies have reported that there was a positive correlation between health behavior and health perceptions [33]. Furthermore, it is argued that help from schools, teachers, and communities is needed to improve the effectiveness of health perception programs for adolescents, and such programs should lead to effective programs in the future [31]. Given that as people get older their interest in health generally tends to increase, adolescents are more inclined to have a relatively low interest in health compared to people in other age groups. Thus, enhancing health perceptions during adolescence can lead to lifelong health habits, and it is necessary to explore a variety of educational strategies to improve health perceptions among adolescents.

Third, sports participation had a positive effect on health promoting behavior. A study comparing the health promoting behavior and sports participation of college students in the Republic of Korea and Japan [14] found that the effect of lifestyle factors on health promoting lifestyles and sports activities in Japan and Korea is different. Furthermore, they investigated health promoting behavior in university sports participants and found results that are consistent with the findings of the present study [18]. In addition, a study reported that the experience of participation in program on physical fitness in Hungarian older adults had a positive effect on health promoting behavior [15]. From these results, it can be inferred that students’ continuous participation in sports activities can effectively affect their health promoting behavior, they can live healthy daily lives, including their schoolwork and school life. Therefore, when deciding on health promoting behavior projects and systems for adolescents, it is necessary for educational institutions, such as school authorities and the Ministry of Education, to consider the results of this study.

Fourth, sports participation had a complete mediating effect in the relationship between health perceptions and health promoting behavior. The present results show that adolescents with higher levels of health perceptions tend to participate more actively in sports activities, which may lead to increased health promoting behavior in adolescents. Active participation in sports activities in adolescence, which is a critical period that can lead to lifelong health, can be seen as a prerequisite for living a healthy life. The results of this study are significant in that they reveal the importance of sports participation as a variable for forming a healthy lifestyle among adolescents. Moreover, we believe these results are a theoretical basis for developing various policies and programs for adolescents’ sports participation.

Recently, because of the pandemic phenomenon caused by COVID-19, the importance of personal hygiene management and a healthy lifestyle is being emphasized. This experience will make it extremely important for individuals to renew their perception of health. Regular participation in sports to improve health from adolescence can be seen as a solid foundation for lifelong health [34,35]. The results of this study will help provide a theoretical foundation for the development and operation of educational institutions’ programs to strengthen awareness of youth health and encourage participation in sports.

## 5. Conclusions

The aim of this study was to verify the structural relationship between health perceptions, sports participation, and health promoting behavior in adolescents. The results of the study are as follows: first, health perceptions were found to have a positive effect on sports participation. Furthermore, health perceptions were found to have no direct effect on health promoting behavior, while sports participation was found to have a positive effect on health promoting behavior. Additionally, sports participation completely mediated the relationship between health perceptions and health promoting behavior.

Based on the findings and limitations of this study, suggestions for further studies are as follows. First, because the participants in this study were limited to Korean adolescents, it is difficult to generalize the findings to the entire population. Therefore, further studies are needed to involve adolescents in various countries to expand the sample size. Second, since adolescents’ health promoting behaviors can appear as a complex process involving various variables, it is necessary to conduct further studies using diversified variables that may affect health promoting behaviors based on the results of this study. Third, since the scales used in this study were based on self-report, there is a possibility that the participants might respond with defensive attitudes, and their responses might be diminished or exaggerated. Therefore, in order to overcome the limitations of the self-report questionnaire, it is necessary to examine various types of tests such as the use of a social desirability scale. Fourth, in this study, the measurement of sports participation consisted of a five-point Likert scale. In further studies, it will be necessary to conduct research on sports participation by degree (strength, frequency, and duration) and examine the differences in health-related eccentricities according to the level of participation.

## Figures and Tables

**Table 1:** General characteristics of the participants.

Variable	Classification	Preliminary Survey		Main Survey		Total	
		Number of Cases	Percentage (%)	Number of Cases	Percentage (%)	Number of Cases	Percentage (%)
School level	Middle school	91	50.56	172	52.60	263	51.87
	High school	89	49.44	155	47.40	244	48.13
Gender	Male	95	52.78	180	55.05	275	54.24
	Female	85	47.22	147	44.95	232	45.76
Total		180	100.00	327	100.00	507	100.00

**Table 2:** Reliability analysis.

Variables		Cronbach’s α
Health perceptions	Importance of health	0.526
	Health interest	0.677
	Confidence in health recovery	0.787
	Health concern	0.564
Health promoting behavior	Personal hygiene, life habits	0.359
	Nutrition & exercise management	0.699
	Eating habit management	0.674
	Stress management	0.630
	Health responsibility	0.619
Sports participation	Cognitive participation	0.917
	Behavior participation	0.895
	Affective participation	0.811

**Table 3:** Goodness-of-fit indexes in confirmatory factor analysis of proposed and revised models.

Models	x^2^/DF	RMR	GFI	IFI	CFI	RMSEA
Proposed model	2.871	0.041	0.886	0.958	0.958	0.110
Revised model	2.198	0.036	0.928	0.976	0.975	0.089

DF = Degree of Freedom, RMR = Root Mean square Residual, GFI = Goodness of Fit Index, IFI = Incremental Fit Index, CFI = Comparative Fit Index, RMSEA = Root Mean Square Error of Approximation.

**Table 4:** Results of confirmatory factor analysis.

Variables			Non-Standardized Coefficient	S.E.	C.R.	*p*	Standardized Coefficient	Construct Reliability	AVE
Health perceptions	→	A	1.000	-	-	-	0.555	0.547	0.951
	→	B	1.323	0.193	6.855	<0.001 ***	0.763		
	→	C	1.484	0.220	6.761	<0.001 ***	0.741		
Health promoting behavior	→	D	1.000	-	-	-	0.705	0.978	0.917
	→	E	0.895	0.121	7.404	<0.001 ***	0.642		
	→	F	1.100	0.148	7.438	<0.001 ***	0.645		
	→	G	1.081	0.139	7.768	<0.001 ***	0.679		
Sports participation	→	H	1.000	-	-	-	0.946	0.985	0.955
	→	I	0.863	0.047	18.264	<0.001 ***	0.888		
	→	J	0.801	0.052	15.515	<0.001 ***	0.819		

S.E. = standard error, C.R. = critical ratio, AVE = average variance extracted; A = importance of health, B = health interest, C = confidence in health recovery, D = nutrition and exercise management, E = eating habit management, F = stress management, G = health responsibility, H = cognitive participation, I = behavioral participation, J = affective participation; *** *p* < 0.001, tested by confirmatory factor analysis.

**Table 5:** Discriminant validity verification.

Variables	Correlations between the Constructs			Average Variance Extracted
	1	2	3	
Health perceptions	1.000	-	-	0.951
Health promoting behavior	0.630 ***	1.000	-	0.917
Sports participation	0.515 ***	0.421 ***	1.000	0.955

*** *p* < 0.001, tested by correlation analysis.

**Table 6:** Descriptive statistical analysis results.

Variables		Mean	Standard Deviation	Skewness	Kurtosis
Health perception	Importance of health	3.83	0.56	−0.367	1.557
	Health interest	3.49	0.53	0.091	0.186
	Confidence in health recovery	3.68	0.68	0.057	−0.314
Health promoting behavior	Nutrition & exercise management	3.37	0.67	0.004	0.205
	Eating habit management	3.04	0.68	0.064	0.047
	Stress management	3.06	0.81	0.096	0.083
	Health responsibility	3.72	0.75	−0.317	−0.122
Sports participation	Cognitive participation	2.89	1.11	0.260	−0.694
	Behavioral participation	2.58	1.03	0.404	−0.565
	Affective participation	3.34	0.99	−0.317	−0.218

**Table 7:** Goodness-of-fit indices for the study model.

Model	x^2^/DF	RMR	GFI	IFI	CFI	RMSEA
Proposed model	3.071	0.035	0.945	0.954	0.954	0.080

DF = Degree of Freedom, RMR = Root Mean Square Residual, GFI = Goodness of Fit Index, IFI = Incremental Fit Index, CFI = Comparative Fit Index, RMSEA = Root Mean Square Error of Approximation.

**Table 8:** Path analysis results.

Path			Standardized Regression Coefficient	Regression Coefficient	S.E.	C.R.	*p*	Testing
Health perceptions	→	Sports participation	0.570	0.235	0.030	7.885	<0.001 ***	Supported
Health perceptions	→	Health promoting behavior	0.045	0.021	0.036	0.592	0.554	Rejected
Sports participation	→	Health promoting behavior	0.749	0.872	0.122	7.159	<0.001 ***	Supported

S.E. = standard error, C.R. = critical ratio; *** *p* < 0.001, tested by path analysis.

**Table 9:** Goodness-of-fit indices of hypothesis and competition models.

Model	x^2^	DF	TLI	CFI	RMSEA
Hypothesis model	98.275	32	0.935	0.954	0.080
Competition model	98.561	33	0.938	0.955	0.078

DF = Degree of Freedom, TLI = Tucker Lewis Index, CFI = Comparative Fit Index, RMSEA = Root Mean Square Error of Approximation; ∆x^2^ = 0.286.

**Table 10:** Indirect effects analysis.

Path					Estimate	S.E.	Bias-Corrected Bootstrap	
							Lower	Upper
Health perception	→	Sports participation	→	Health promoting behavior	0.454	0.051	0.355	0.551

S.E. = standard error, tested by bootstrapping method.
